# Magnetic Resonance Imaging (MRI) Findings in COVID-19 Associated Encephalitis

**DOI:** 10.3390/neurolint15010005

**Published:** 2023-01-05

**Authors:** Manoj Tanwar, Aparna Singhal, Mohammadreza Alizadeh, Houman Sotoudeh

**Affiliations:** 1 Department of Radiology, Heersink School of Medicine, University of Alabama at Birmingham, Birmingham, AL 35233, USA; 2 Physiology Research Center, Iran University of Medical Sciences, Tehran 14665-354, Iran

**Keywords:** COVID-19, magnetic resonance imaging, encephalitis

## Abstract

We conducted this study to investigate the scope of the MRI neuroimaging manifestations in COVID-19-associated encephalitis. From January 2020 to September 2021, patients with clinical diagnosis of COVID-19-associated encephalitis, as well as concomitant abnormal imaging findings on brain MRI, were included. Two board-certified neuro-radiologists reviewed these selected brain MR images, and further discerned the abnormal imaging findings. 39 patients with the clinical diagnosis of encephalitis as well as abnormal MRI findings were included. Most (87%) of these patients were managed in ICU, and 79% had to be intubated-ventilated. 15 (38%) patients died from the disease, while the rest were discharged from the hospital. On MRI, FLAIR hyperintensities in the insular cortex were the most common finding, seen in 38% of the patients. Micro-hemorrhages on the SWI images were equally common, also seen in 38% patients. FLAIR hyperintensities in the medial temporal lobes were seen in 30%, while FLAIR hyperintensities in the posterior fossa were evident in 20%. FLAIR hyperintensities in basal ganglia and thalami were seen in 15%. Confluent FLAIR hyperintensities in deep and periventricular white matter, not explained by microvascular angiopathy, were detected in 7% of cases. Cortical-based FLAIR hyperintensities in 7%, and FLAIR hyperintensity in the splenium of the corpus callosum in 7% of patients. Finally, isolated FLAIR hyperintensity around the third ventricle was noted in 2% of patients.

## 1. Introduction

SARS-CoV-2 outbreak was first reported in Wuhan and has continued to cause devastation across the globe, with more than 6.3 million reported deaths to date [1]. New strains have emerged which have infected even vaccinated persons. Respiratory, hematologic, cardiac, and gastrointestinal complications of COVID-19 have been extensively studied. Although COVID-19 primarily affects the respiratory system, studies have found that it also adversely impacts the neurological system. The most commonly reported neurological symptoms include fatigue, myalgia, taste alteration, smell impairment, and headache [2,3]. Central nervous system (CNS) manifestations include stroke, cerebrovascular thromboembolism, anosmia, and myelitis. Thrombogenic complications of the disease, such as ischemic stroke and dural venous thrombosis, are well-known [4]. Limited studies and case reports have also reported non-hemorrhagic and hemorrhagic leukoencephalitis as potential complications of COVID-19 [5].

Encephalitis is defined as CNS inflammation mediated by viral and, less commonly, bacterial infectious agents or immune reactions, and presents with symptoms of altered mental status (AMS), seizures, and fever [6]. Encephalitis is a rare manifestation of COVID infection with an incidence of 0.2–1%, but a poor prognosis. It has been reported that the incidence of COVID encephalitis in severely ill patients is about 6–7% [7]. Encephalitis usually presents 14 days after the onset of COVID symptoms. About 83% of patients with encephalitis are already admitted to the intensive care unit (ICU). The mean age of COVID-associated encephalitis is 59 years without a gender preference. Hypertension, hyperlipidemia, and diabetes mellitus are the most common comorbidities. The most frequent manifestation of COVID-associated encephalitis is decreased level of consciousness, altered mental status, seizures, headaches, and weakness [7]. The mechanism of COVID-associated encephalitis is not known. Possible mechanisms of CNS inflammation in these patients include autoimmune reaction to the anti-NMDA (N-methyl D-aspartate) receptor antibodies against the glutamatergic pathways [8], inflammation of the glial system [9,10], or ischemic changes caused by intravascular thrombosis given the presence of ACE2 receptors in the vascular endothelium [11]. Therefore, diagnosing COVID-19-induced encephalitis remains a challenge due to a variable range of symptoms, neuroimaging findings, and lack of an established diagnostic criteria. Detection of SARS-CoV2 in the CSF remains insufficient [12]. 

Therefore, this retrospective study explored the spectrum of the neuroimaging manifestations of COVID-associated encephalitis. 

## 2. Materials and Methods

This study was approved by the local ethical committee of our tertiary care institution (IRB300008377-0022, 8 November 2021) in United States. After scrutinizing the radiology reports for magnetic resonance imaging (MRI) studies performed on COVID-19 patients from January 2020 to September 2021, all patients with clinical diagnosis of COVID-associated encephalitis were included. Exclusion criteria included non-COVID-19-encephalitis pathologies, such as acute or chronic infarcts, intracranial malignancy, and known encephalitis due to other causes such as infection, metabolic derangements, and inflammatory causes. The diagnosis of COVID-19 encephalitis was established using modified diagnostic criteria combining the clinical diagnosis by the primary care team, diagnostic criteria for encephalitis [13] (Table 1), and a mandatory presence of positive COVID-19 polymerase chain reaction (PCR) test. Patients with an encephalitis diagnosis from the primary care team, however, not meeting the criteria based on the diagnostic criteria for encephalitis were excluded. All MRI imaging was performed on 3 T and 1.5 T Siemens and Philips MRI scanners at our university hospital affiliated with a medical school. Patients’ clinical information, such as age, gender, co-morbidities, presence of AMS, fever, seizure, EEG findings, initial presenting symptoms, presence of neurological deficits or psychiatric symptoms, reason for MRI, and days from admission to MRI were recorded from the Cerner powerchart (Kansas City, Missouri). Two board-certified neuro-radiologists then reviewed the brain MRIs of the selected patients regarding the presence of abnormal imaging findings. Both neuroradiologists being at separate workstations simultaneously reviewed these cases over Zoom video conference (San Jose, California), using Philips IntelliSpace PACS (Amsterdam, The Netherlands) to ensure appropriate social distancing during the pandemic. Image findings were recorded after consensus. T1-weighted images were utilized for screening structural abnormalities. We reviewed the FLAIR (fluid-attenuated inversion recovery), SWI (susceptibility-weighted images), DWI (diffusion-weighted images), ADC (apparent diffusion coefficient), and post-contrast (if available) MRI sequences for any abnormal findings of hemorrhagic or non-hemorrhagic encephalitis. Since the majority of patients did not undergo contrast enhanced imaging, we excluded the post-contrast imaging findings. Studied findings include microhemorrhages, macroscopic hemorrhages, and FLAIR hyperintensities. FLAIR signal characteristics of the bilateral frontal lobe cortices were used as a comparative standard for assessing the FLAIR hyperintensity. DWI images were primarily utilized for the exclusion of acute and subacute infarcts. In addition, we also recorded the location of the abnormality: medial temporal lobe, other specific parenchymal lobes, diffuse presence, insula, basal ganglia, thalamus, corpus callosum, periventricular location, middle cerebellar peduncle, cerebellum, and brainstem. Furthermore, we also recorded ICU admission, need of ventilation, treatment received, days in the hospital, and final outcome for all participants.

## 3. Results

**Figure 1:**
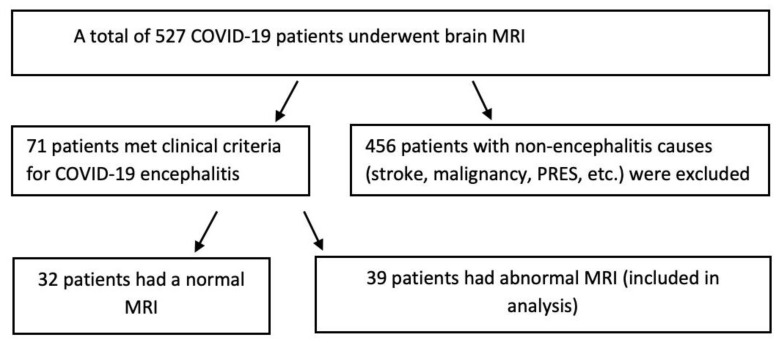
Patient enrollment flow chart.

During the study time frame, a total of 527 COVID-19 patients underwent brain MRI, from which patients with stroke, malignancy, PRES, and other non-COVID-19 encephalitis pathologies were excluded. 71 patients met the clinical criteria of COVID-19 encephalitis, out of which 32 patients had a normal brain MRI. Eventually, 39 patients with abnormal brain MRI findings (21 female and 18 male; mean age 61 years) were selected for the final analysis (Figure 1).

The clinical characteristics of these patients are described in Table 2. The most common initial presenting symptoms were fever, cough, shortness of breath, altered mental status, and loss of consciousness. The majority of patients did not report psychiatric symptoms, however, 10/39 patients (26%) reported neurologic deficits. 34/39 (87%) patients were admitted to the intensive care unit (ICU), and 31/39 (79%) had to be intubated-ventilated. MRI scan was performed between 1–90 days after admission (mean = 16 days), while hospital stay ranged from 4–150 days (mean = 36 days). 18 (46%) patients received remdesivir, while 2 (5%) were given acyclovir. A total of 15 (38%) patients died from the disease, while the rest were discharged from the hospital.

**Figure 2:**
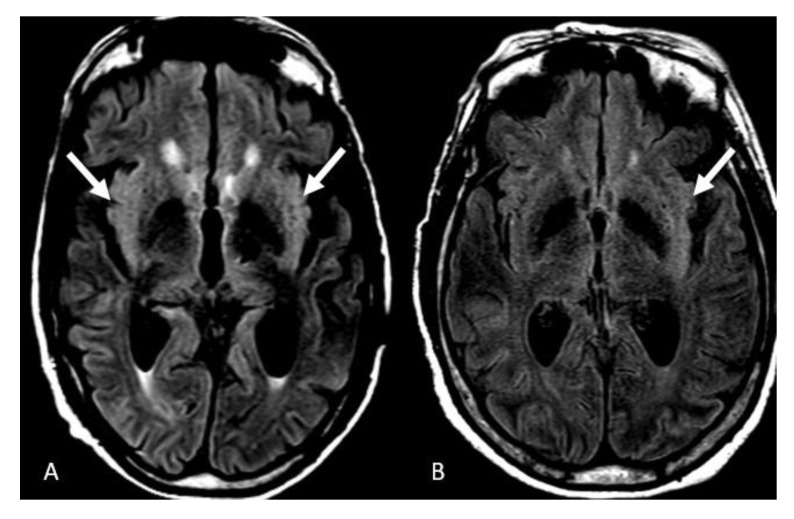
Insular involvement. (**A**) Axial FLAIR images demonstrate bilateral FLAIR hyperintensities in the insular cortex. (**B**) isolated left insular involvement. Image has been windowed manually to improve the visibility of signal abnormality.

**Figure 3:**
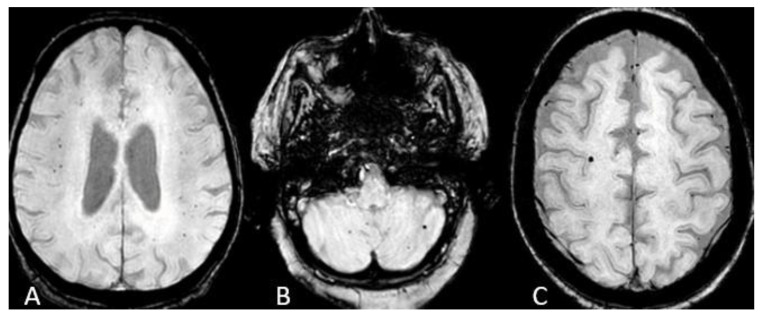
Microhemorrhages. (**A**) SWI images show the diffuse pattern of microhemorrhages. (**B**) Isolated cerebellar microhemorrhages. (**C**) Isolated frontal microhemorrhage.

The imaging characteristics are described in Table 3. FLAIR hyperintensities in the insular cortex were the most common neuroimaging manifestation of the COVID-induced encephalitis, seen in 15 patients (38%), with a bilateral pattern evident in ten of them (25%) (Figure 2A). Isolated left insular involvement was seen in 5 patients (12)% (Figure 2B). We did not detect isolated right insular involvement. Cerebral microhemorrhages on the SWI sequence were equally prevalent manifestation in 15 patients (38%), with a diffuse pattern in 9 patients (23%) (Figure 3A), cerebellar micro-hemorrhages in 2 patients (5%) (Figure 3B), frontal micro-hemorrhages in 2 patients (5%) (Figure 3C), 1 each in left basal ganglia (2%) and corpus callosum splenium (2%).

**Figure 4:**
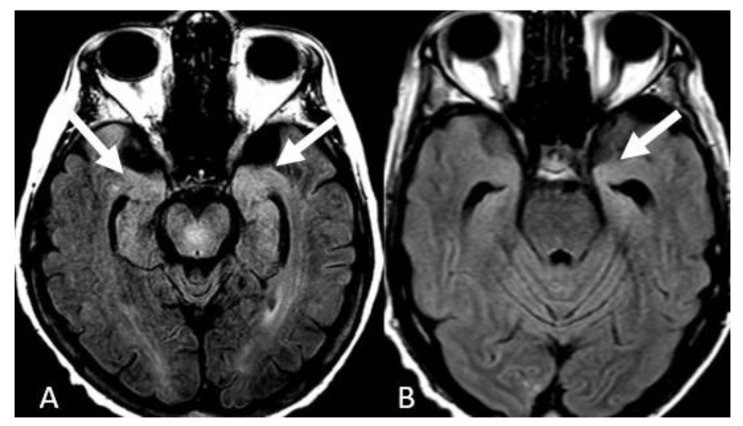
Temporal lobe involvement. (**A**) Axial FLAIR images depict bilateral medial temporal lobe FLAIR hyperintensities. (**B**) Isolated left medial temporal FLAIR hyperintensities. Image has been windowed manually to improve the visibility of signal abnormality.

**Figure 5:**
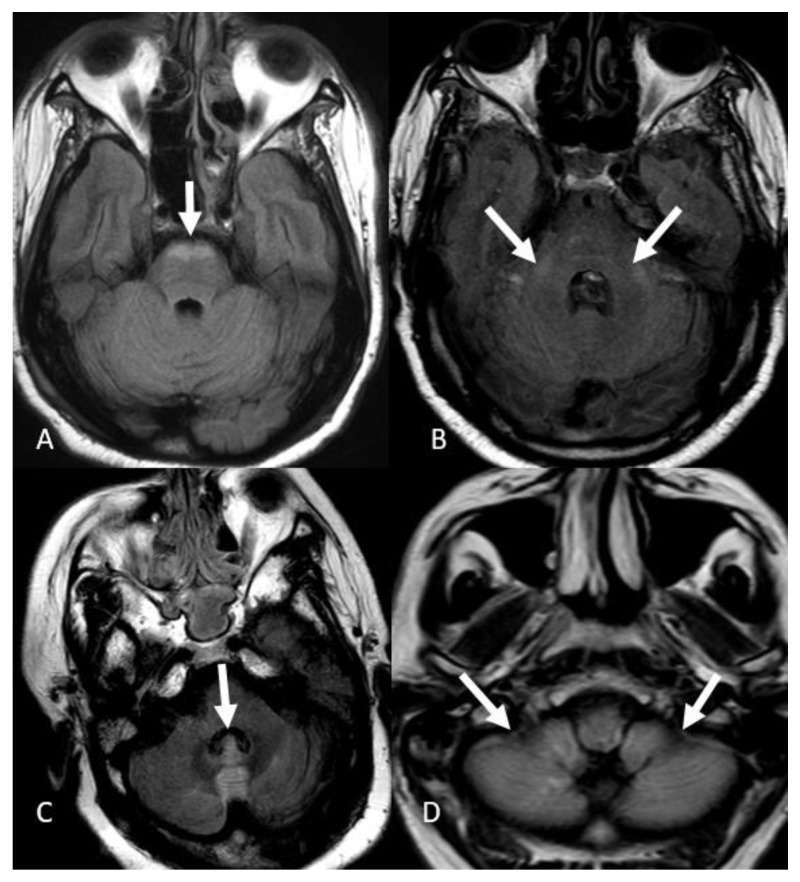
Posterior fossa involvement. (**A**) Axial FLAIR images demonstrate FLAIR hyperintensity in the ventral portion of the pons. (**B**) Bilateral middle cerebellar peduncle FLAIR hyperintensities. (**C**) FLAIR hyperintensity of the vermis. (**D**) FLAIR hyperintensities in the bilateral cerebellar hemispheres. Image has been windowed manually to improve the visibility of signal abnormality.

**Figure 6:**
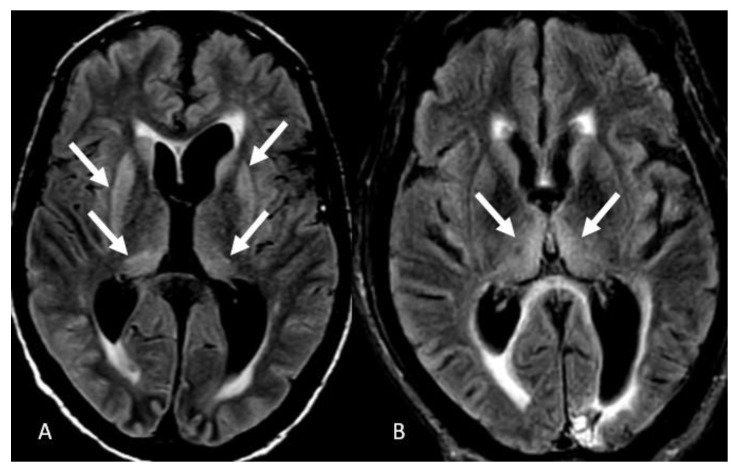
Basal ganglia and thalami involvement. (**A**) Axial FLAIR images show bilateral basal ganglia and thalami FLAIR hyperintensities. (**B**) Bilateral thalami FLAIR hyperintensities. Image has been windowed manually to improve the visibility of signal abnormality.

FLAIR hyperintensities in the medial temporal lobes were the third most common presentation reported in 12 patients (30%). These findings were bilateral in 10 patients (25%) (Figure 4A) and isolated left-sided in 2 patients (5%) (Figure 4B). We did not detect isolated right temporal FLAIR hyperintensities. FLAIR hyperintensities in the posterior fossa were the fourth most common manifestation of COVID-19-associated encephalitis evident in 9 patients (20%), which in 6 of them effected the ventral pons (15%) (Figure 5A), bilateral middle cerebellar peduncles in 1 patient (Figure 5B), vermis in 1 patient (Figure 5C), and bilateral cerebellar hemispheres in 1 patient (Figure 5D). FLAIR hyperintensities in the basal ganglia and thalami were the fifth most common finding evident in 6 patients (15%), with a bilateral involvement pattern in 4 patients (10%) (Figure 6A), and isolated bilateral thalami findings in 2 patients (5%) (Figure 6B).

**Figure 7:**
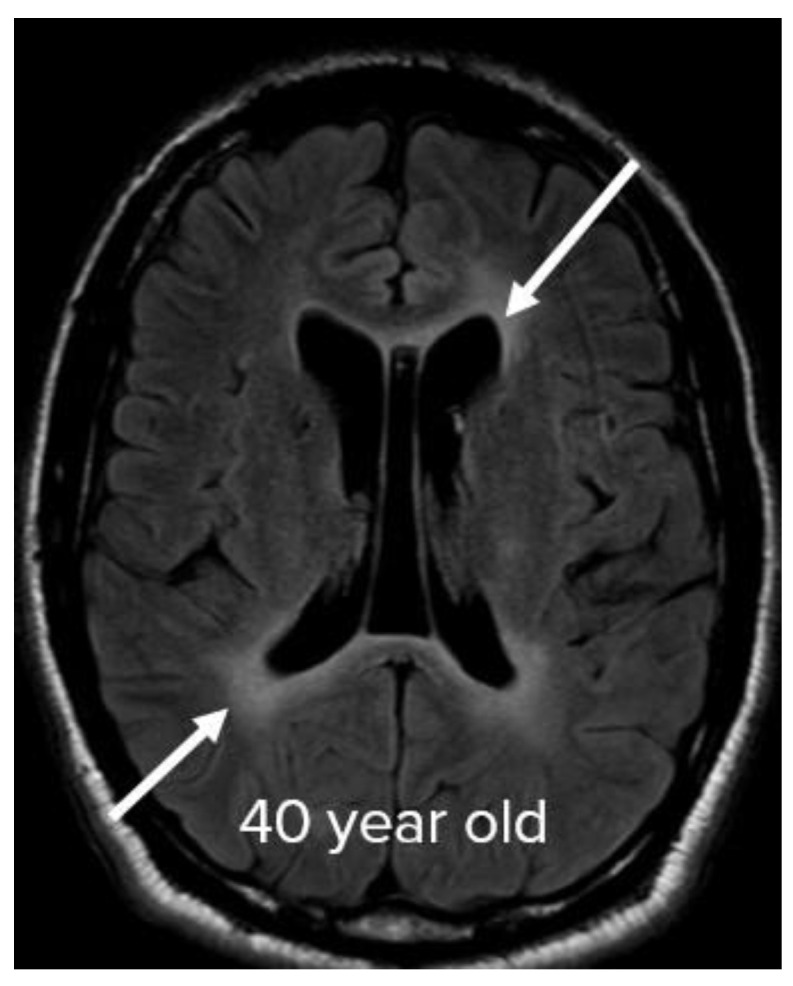
Periventricular involvement. Axial FLAIR image depicts periventricular FLAIR hyperintensities in a 40-year-old patient, otherwise not explained by chronic microangiopathic changes and absence of other risk factors. Image has been windowed manually to improve the visibility of signal abnormality.

**Figure 8:**
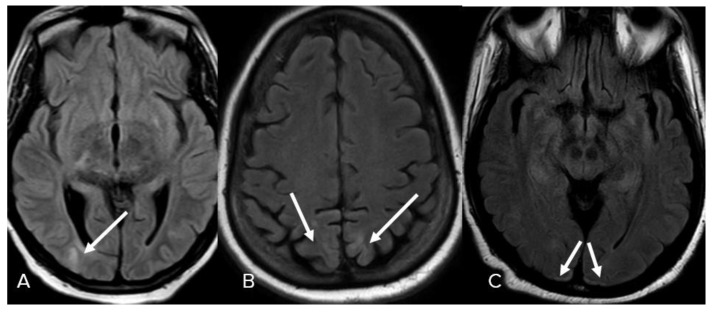
Cortical involvement. (**A**) Axial FLAIR images demonstrate isolated cortical-based FLAIR hyperintensity in the right occipital lobe. (**B**) Bilateral parietal FLAIR hyperintensities. (**C**) Bilateral occipital FLAIR hyperintensities. Image has been windowed manually to improve the visibility of signal abnormality.

**Figure 9:**
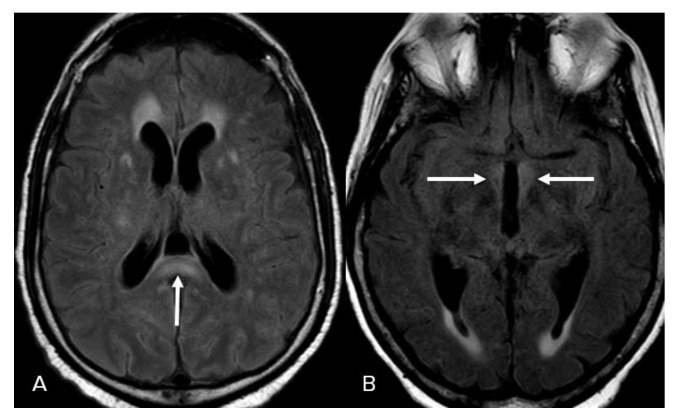
Less common patterns. (**A**) Axial FLAIR images show FLAIR hyperintensity of the splenium of the corpus callosum. (**B**) FLAIR hyperintensity around the third ventricle. Image has been windowed manually to improve the visibility of signal abnormality.

In some cases, confluent patchy FLAIR hyperintensities in the deep and periventricular white matter (diffuse leukoencephalopathy) could not be explained by chronic microvascular angiopathic changes, and were considered to be the sixth manifestation in 3 patients (7%) (Figure 7). Cortical-based FLAIR hyperintensities were seen in 3 patients (7%), involving the right occipital lobe in one of the patients (2%) (Figure 8A), bilateral parieto-occipital lobes in 1 patient (2%) (Figure 8B), and bilateral occipital lobes in 1 patient (2%) (Figure 8C). Less common patterns include FLAIR hyperintensities in the splenium of the corpus callosum observed in 3 patients (7%). (Figure 9A). Finally, isolated FLAIR hyperintensities around the third ventricle were seen in 1 patient (2%) (Figure 9B).

## 4. Discussion

We conducted this study to navigate the unknown scope of neuroimaging manifestations in COVID-associated encephalitis. After extensive research on the patient’s medical records, 39 patients with a mean age of 61 years were enrolled in the study. The results demonstrated that abnormal FLAIR hyperintensities in the insular cortex were the most common radiologic findings in COVID-associated encephalitis, reported in 38% of the patients. In addition, micro-hemorrhages evident on the SWI sequences were equally prevalent. Moreover, FLAIR hyperintensities in the medial temporal lobes were observed in 30%, which was the third most common presentation. It is also worth noting that FLAIR hyperintensities in the posterior fossa, basal ganglia and thalami accounted for 20% and 15% of cases, respectively. Finally, this data suggests that diffuse leukoencephalopathy, abnormal signal intensity in the splenium of the corpus callosum, and periventricular white matter signal changes around the third ventricle may be the other less common manifestations.

Our results are consistent with Kandemirli et al. study, which suggested that one of the most common imaging finding was cortical signal abnormalities on FLAIR images [14]. Presence of FLAIR abnormalities and microhemorrhages has been reported in other studies as well [5,15,16,17]. Some studies have proposed leptomeningeal enhancement; however, majority of our patients did not undergo contrast-enhanced images. Furthermore, some articles have described the presence of restriction diffusion in the white matter; however, we primarily utilized DWI images to exclude cases with acute and subacute infarcts to avoid overlap with ischemic changes secondary to thrombotic complications [18,19]. 

MRI findings in critically ill or ICU-admitted COVID-19 patients have been previously published by many researchers. The precise pathophysiology is unclear; however, authors have hinted towards immunologic parainfectious processes [20], which has been supported by some neuropathologic studies [21]. Radmanesh et al. evoked the assumptions of hypoxia or small-vessel vasculitis [16]. Direct brain tissue involvement by viral particles has not been endorsed by cerebrospinal fluid analysis, and other hypotheses such as postinfectious demyelinating diseases may be considered. Since most of these patients were admitted to intensive care units for acute respiratory distress syndrome, other assumptions include delayed post hypoxic leukoencephalopathy [22], metabolic or toxic encephalopathy, and posterior reversible encephalopathy syndrome [23]. 

While differentiation from other causes of encephalitis can be difficult, other etiologies such as herpes encephalitis present with a fulminant course, with 90% of cases resulting from HSV-1. Diagnosis is established with CSF PCR, although a combination of the clinical scenario, CSF pleocytosis and elevated protein, and appropriate imaging are usually highly suggestive and permit commencement of treatment [24]. Similarly, autoimmune encephalitis can present with the involvement of the mesial temporal lobes and limbic system, manifested by cortical thickening and increased T2/FLAIR signal intensity. However, in approximately 60% of cases, antineuronal antibodies are present, such as the anti-Hu antibody in small cell lung cancer, anti-Ta antibody in testicular cancers, anti-NMDA NR1 in ovarian teratomas, or anti-NMDA NR2 in SLE patients [25]. 

The current study is one of the most extensive research studies by sample size, assessing imaging findings of clinically documented encephalitis in COVID-19 patients. However, we may have encountered some limitations. The study’s retrospective nature may affect its reliability in some cases and limit prospective application. Additionally, although this study was conducted at a large tertiary care center, pooling data from multiple institutions will add to the generalizability of the results. Additionally, post-contrast sequences were not evaluated in our study but may reveal additional useful imaging features in COVID-associated encephalitis. As a result, further multi-institutional investigations and also utilization contrast-enhanced imaging can be considered for future studies. 

## 5. Conclusions

COVID-associated encephalitis manifests as a spectrum of neuroimaging findings on brain MRI. The most common manifestations are abnormal FLAIR hyperintensities in the insular cortex, and diffuse or focal microhemorrhages. Other neuroimaging manifestations are abnormal FLAIR hyperintensities in the medial temporal lobes, brainstem, abnormal cortical hyperintensities, and abnormal hyperintensities within the thalami and basal ganglia. Other less common findings include diffuse leukoencephalopathy, abnormal FLAIR hyperintensities in the corpus callosum splenium, and around the third ventricle.

## Figures and Tables

**Table 1:** Modified Diagnostic Criteria for COVID-19 Encephalitis [13].

**Modified Diagnostic Criteria for COVID-19 Encephalitis**
**Major Criterion (required)**
Patient must have a positive COVID-19 PCR test
Patients presenting to medical attention with altered mental status (defined as decreased or altered level of consciousness, lethargy or personality change) lasting >= 24 h with no alternative cause identified
**Minor Criteria (2 required for possible encephalitis; >= 3 required for probable or confirmed encephalitis)**
Documented fever >= 38 C (100.4 F) within the 72 h before or after presentation
Generalized or partial seizures not fully attributable to a preexisting seizure disorder
New onset of focal neurologic findings
CSF WBC count >= 5/cubic mm
Abnormality of brain parenchyma on neuroimaging suggestive of encephalitis that is either new from prior studies or appears acute in onset
Abnormality on electroencephalography that is consistent with encephalitis and not attributable to another cause
Abbreviations: CSF-cerebral spinal fluid, PCR-polymerase chain reaction, WBC-white blood cell

**Table 2:** Clinical Characteristics of the Participants.

ID	Age	Sex	Comorbidities	AMS > 24 h	T >= 38, 100.4 F	Generalized or Partial Seizure	EEG	Initial Presenting Symptoms	Neural Deficits	Psychiatric Symptoms	Admission to MRI (in Days)	Reason for MRI	ICU Admission	Ventilated?	Treatment	Days in Hospital	Outcome
1	51	M	Hemorrhagic stroke, HTN, DM, BPH, HLD, gout and impaired mobility	yes	yes	yes	yes	AMS, encephalitis	no	no	3	Encephalitis and seizure	yes	yes	Dexamethasone, remdesivir	27	Discharge
2	42	F	ESRD on PD, CVA, DM2, and blindness	yes	yes	no	no	Fever, cough, weakness	yes	no	1	Confusion	yes	yes	Dexamethasone	28	Death
3	49	M	Type II DM	yes	yes	yes	yes	Fever, cough, AMS	no	no	19	Encephalitis	yes	yes	Dexamethasone	38	Discharge
4	82	F	Type I diabetes mellitus, hypertension	yes	yes	yes	yes	Confusion, acute onset aphasia, encephalitis	yes	no	1	Aphasia, encephalitis, facial droop, visual field loss	yes	yes	Dexamethasone, remdesivir	17	Discharge
5	62	F	Renal transplant, recurrent UTIs, CKD, HTN, DM	yes	yes	no	no	AMS	no	no	9	AMS, encephalitis	yes	yes	Dexamethasone	17	Death
6	68	F	NASH cirrhosis, hepatic encephalopathy, DM, HLD, pancreatitis, OSA, HTN	yes	yes	no	no	Shortness of breath, cough, confusion	no	no	13	Encephalitis, AMS	yes	yes	Dexamethasone, remdesivir	28	Discharge
7	62	M	HTN, type II DM, OSA, rectal fissure, inhalation injury	yes	yes	yes	no	Fatigue, weakness, cough, dyspnea,	no	no	61	Encephalitis	yes	yes	Dexamethasone, remdesivir	65	Discharge
8	68	M	Uncontrolled HTN, vascular dementia	yes	yes	no	no	Hypertensive emergency	no	no	7	Encephalitis and Parkinsonian symptoms	yes	yes		57	Discharge
9	59	M	Morbid obesity, NIDDM, HTN, gout, GERD/PUD, OSA, retinal vein occlusion	yes	yes	yes	yes	Fever, cough, progressive dyspnea	yes	no	36	Encephalitis, hydrocephalus	yes	yes	Dexamethasone, remdesivir	49	Discharge
10	59	F	DVT (on Xarelto), HTN, sickle cell disease, CKD	yes	yes	no	yes	Syncopal episode with residual weakness, L foot and neck pain	yes	no	6	Encephalitis, follow up stroke	yes	yes	Dexamethasone	15	Discharge
11	53	F	DM type I, renal transplant, neuropathy, hypothyroidism, HTN	yes	yes	no	no	Generalized body aches, decreased UOP and fever	no	no	10	Encephalitis	yes	yes	Dexamethasone, remdesivir	46	Discharge
12	84	F	A-fib and HTN	yes	yes	yes	yes	Shortness of breath, cough	no	no	9	AMS, possible infarcts seen on CT	yes	yes	Dexamethasone, remdesivir	39	Discharge
13	66	F	HTN, undiagnosed diabetes	yes	yes	yes	yes	AMS, unresponsiveness	yes	no	4	Encephalitis, AMS	yes	no	Dexamethasone, remdesivir	28	Discharge
14	65	F	HTN, diabetes, hyperlipidemia	yes	yes	yes	yes	Shortness of breath, encephalitis	yes	no	23	Encephalitis, AMS, PRES	yes	no	Dexamethasone, remdesivir	36	Discharge
15	42	F	IDDM, HTN	yes	yes	yes	yes	Generalized tonic–clonic seizures	no	no	2	Encephalitis, AMS, seizure	yes	yes	Dexamethasone	6	Death
16	77	F	HTN, gout	yes	yes	no	no	AMS, falls	no	no	5	Encephalitis, confusion	yes	no	Dexamethasone, remdesivir	39	Death
17	78	M	CAD s/p CABG, Parkinson’s L STN stimulator lead, HLD, HTN	yes	yes	yes	yes	Cough, progressive dyspnea, pleuritic chest pain	yes	no	25	Encephalitis	yes	yes	Dexamethasone, remdesivir	34	Death
18	78	F	psychotic disorder, HTN, diabetes	yes	yes	no	no	Hypoxia and lethargy	yes	no	10	AMS	yes	yes	Dexamethasone	24	Discharge
19	32	F	Insulin-dependent diabetes, heart failure, ESRD	yes	yes	yes	yes	Shortness of breath, cough and fever.	no	no	6	Status post PEA arrest	yes	yes	Dexamethasone	14	Death
20	58	F	Hypertension, diabetes, schizophrenia, hepatitis C	yes	yes	no	yes	Dyspnea, progressive lethargy, fever, loss of appetite and hypotension	no	no	21	Encephalitis, AMS	yes	yes	Dexamethasone, remdesivir	30	Discharge
21	82	F	HTN, DM	yes	yes	yes	yes	Loss of consciousness	no	no	9	AMS	yes	yes	Dexamethasone, remdesivir	16	Death
22	77	M	CAD, aortic valve stenosis	yes	yes	no	yes	Hypoxia after surgery for TAVR	no	no	36	Encephalitis, AMS	yes	yes		59	Death
23	60	F	HLD, DM, stroke, AML, migraine, depression, spinal stenosis	yes	yes	no	yes	Loss of consciousness	no	no	10	AMS, weakness	yes	yes		37	Discharge
24	41	M	MS	yes	yes	no	yes	Fever, blurred vision	yes	no	1	Ataxia, blurred vision	no	no		5	Discharge
25	66	F	Sarcoidosis, lung transplant	yes	yes	no	yes	Shortness of breath	no	no	30	Encephalitis	no	no	Dexamethasone, remdesivir	40	Death
26	55	F	HTN, DM, CSF leak, gout, fibromyalgia	yes	yes	yes	yes	Fever, cough, AMS	no	no	7	Encephalitis	no	no	Dexamethasone	20	Discharge
27	45	M	HTN, CKD, obesity	yes	yes	no	yes	Hypoxia	no	no	17	Encephalitis	yes	yes	Dexamethasone, remdesivir	56	Discharge
28	46	M	Obesity, gout,	yes	yes	no	yes	Hypoxia	no	no	21	Encephalitis	yes	yes	Acyclovir	43	Death
29	69	F	COPD, sarcoidosis, schizoaffective, DM, HTN	yes	yes	no	yes	Fever	no	no new	13	Encephalitis	yes	yes	Dexamethasone, remdesivir	26	Death
30	41	M	None	yes	yes	no	yes	Weakness	no	no	23	AMS	yes	yes	Dexamethasone, remdesivir	31	Death
31	41	F	CHF, DM, GERD,	yes	yes	no	yes	Fever	no	no	90	Encephalitis	yes	yes		150	Death
32	56	M	CVA, TIA, depression, anemia, HTN, DM	yes	yes	yes	yes	Seizure	no	no	2	Encephalitis, seizure	yes	yes	Acyclovir	5	Discharge
33	72	M	DM, DAC,	yes	yes	no	yes	Hypoxia	no	no	34	Encephalitis	yes	yes		45	Death
34	77	M	HTN, DM, CHF, HTN, prostate CA	yes	no	no	yes	Hypoxia	no	no	12	Encephalitis	yes	yes		47	Discharge
35	61	M	HTN, TBI	yes	no	yes	yes	Seizure	yes	no	13	Seizure	no	no	Dexamethasone, remdesivir	90	Discharge
36	63	M	DM, COPD, ESRD	yes	yes	yes	yes	Hypoxia	no	no	18	AMS	yes	yes	Dexamethasone	24	Death
37	83	M	HTN, HLD, DM	yes	no	yes	yes	Loss of consciousness	no	no	4	AMS	no	no		4	Discharge
38	47	F	DM, HTN, obesity	yes	yes	no	yes	Hypoxia	no	no	9	AMS	yes	yes		45	Discharge
39	68	M	DM, HTN, obesity	yes	no	no	yes	Shortness of breath	no	no	23	Encephalitis	yes	yes		33	Discharge

**Table 3:** Imaging Characteristics of the Participants.

ID	Age	Sex					FLAIR Hyperintensities					Microhemorrhages
			Insula	Medial Temporal Lobe	Ventral Pons	Cerebellum	Deep Gray Matter	Cortical Signal Abnormality	Confluent Leukoencephalopathy	Around Third Ventricle	Corpus Callosum Splenium	
1	51	M			Yes							
2	42	F						Right occipital				
3	49	M	B/l	B/l	Yes			B/l occipital and pareital				Cerebellar
4	82	F	Left									
5	62	F	Left									Right frontal, right cerebellum
6	68	F	B/l	B/l	Yes		B/l basal ganglia & thalami					
7	62	M					B/l basal ganglia & thalami					
8	68	M										Multiple
9	59	M			Yes							
10	59	F							Yes			
11	53	F	B/l		Yes							
12	84	F	B/l	B/l								
13	66	F										Multiple
14	65	F	B/l	B/l								
15	42	F					B/l basal ganglia & thalami					
16	77	F										Multiple
17	78	M	B/l	B/l			B/l thalami					
18	78	F								Yes	Yes	
19	32	F										Left basal ganglia
20	58	F							Yes			
21	82	F									Yes	
22	77	M					B/l basal ganglia & thalami					Frontal
23	60	F	B/l	B/l								
24	41	M		B/l	Yes				Yes			
25	66	F										Multiple
26	55	F						B/l parietal				
27	45	M	B/l	B/l								
28	46	M				Vermis						
29	69	F	Left									Multiple
30	41	M		Left								Corpus callosum splenium
31	41	F	B/l	B/l								
32	56	M				B/l						Multiple
33	72	M										Multiple
34	77	M			B/l middle cerebellar peduncles						Yes	
35	61	M	B/l	B/l								
36	63	M					B/l thalami					Multiple
37	83	M	Left									
38	47	F										Multiple
39	68	M	Left	Left								

Abbreviations: B/l—bilateral.

## Data Availability

The clinical, imaging findings data and anonymized images are already published in the manuscript. Source MRI images belong to specific patients, which are stored in the local radiology PACS, and are not available for public sharing. Any further inquiry can be directed to the authors.

## Abbreviations

ADC = apparent diffusion coefficient, ACE = angiotensin-converting enzyme, AMS = altered mental status, CNS = central nervous system, COVID-19 = coronavirus disease 2019, DWI = diffusion-weighted images, FLAIR = fluid-attenuated inversion recovery, ICU = intensive care unit, GCS = Glasgow coma scale, MRI = magnetic resonance imaging, NIHSS = NIH stroke scale, NMDA = N-methyl D-aspartate, PACS = picture archiving and communication system, PCR = polymerase chain reaction, SARS-CoV-2 = severe acute respiratory syndrome coronavirus 2, SWI = susceptibility-weighted images.
